# Construction of Ecological Security Patterns Based on Circuit Theory under the Resistance Distance Principle

**DOI:** 10.3390/ijerph19106298

**Published:** 2022-05-22

**Authors:** Jinzhao Chen, Zhixiong Mei, Bin Wang, Junchao Wei

**Affiliations:** School of Geography, South China Normal University, Guangzhou 510631, China; jinzhaochen97@163.com (J.C.); wangbinbin_711@163.com (B.W.); weijunchao1997@163.com (J.W.)

**Keywords:** ecological security patterns, circuit theory, resistance distance, Pearl River Delta

## Abstract

Against the background of China’s advocating ecological civilisation construction, an urgent task and a major challenge are to identify key places for ecological protection and restoration and then propose optimisation strategies for future land use, especially in the Pearl River Delta (PRD), one of the regions in China that has the highest urbanisation level. In this study, we find the key places by constructing ecological security patterns and proposing optimisation strategies for future land use by analysing land-use status. We also propose a source identification method based on the resistance distance principle. Results show that forty-six sources were mainly distributed in the mountainous areas surrounding PRD but were less distributed along both sides of the Pearl River estuary. The difference in the spatial distribution of sources is remarkable. Eighty-four corridors generally had spider-like shapes. In the central plain of PRD, corridors were relatively long and narrow. Ninety pinch points were concentrated on existing rivers. Three barriers were located in the corridors between adjacent sources. Two artificial corridors were proposed to be established, which can improve the ecological network connectivity. The method for extracting sources based on the resistance distance principle is proven to be advantageous for improving the integrity of source extraction results and making ecological security patterns more reasonable.

## 1. Introduction

To support the rapid growth of urbanisation, many countries and regions throughout the world have expanded their construction land but occupied ecological land, which has resulted in problems such as habitat loss, ecological network breakage, and ecosystem service value decline, which, in turn, have been seriously threatening regional ecological security [1,2,3]. Ecological security patterns are potential spatial patterns in the landscape, which are composed of some key local positions and spatial connections [4]. In recent years, it has become an effective way to ensure urban ecological security and maintain sustainable development, which comprehensively considers the interaction between ecological processes and landscape patterns. Furthermore, ecological security patterns have been listed among China’s three main strategic patterns of protection and development of China’s territorial space and have become a long-term strategy for coordinating the economic development and ecological protection of China.

At present, the construction of ecological security patterns has formed a paradigm that includes source identification, resistance surface construction, and corridor extraction. The term ‘ecological source’ refers to patches with high ecosystem service value, stable ecological function, and certain continuity [5]. Ecological sources can be identified through the following three main ways: (1) select green areas, water, nature reserves or large area of forest as ecological sources directly [6,7,8,9]; (2) extract the ecological source area by using morphological spatial pattern analysis (MSPA) based on land use and morphological analysis [10]; (3) establish a comprehensive evaluation system to identify the source. The first approach is more subjective. The second approach (MSPA) solves the problem of subjective interference of the first approach and attaches importance to the landscape connectivity of patches, which increases the rationality of ecological sources. However, this method relies on land use data only and ignores spatial heterogeneity without evaluating the functional attributes of ecological land. The comprehensive evaluation method can evaluate the functional properties of ecological land, such as soil conservation capacity, water resource conservation capacity, carbon fixation capacity, and biodiversity [11,12,13], while at the same time, it also considers the connectivity importance of ecological land in the landscape [14]. In general, this method requires screening preselection sources according to the size of sources to ensure that the ecological function radiation range of the selected source is large and that the number of sources is under control, thereby facilitating the improvement of the efficiency of ecological corridor extraction models. However, this process will destroy the integrity of sources because many small sources that are adjacent yet not contiguous to important sources are abandoned in an incogitant manner.

Resistance surface construction is the key preliminary task of corridor extraction through ecological sources. The value of the resistance surface describes the difficulty of ecological processes in heterogeneous landscapes, including species migration and energy flow. A great resistance value corresponds to a great degree of human influence that biological organisms encounter when passing here. Habitat quality refers to the capability of an ecosystem to provide the necessary resources and conditions for all its wildlife or specific populations [15]. A high habitat quality means a high level of biodiversity and a low level of ecological resistance to species; thus, the reciprocal of habitat quality can be used to measure ecological resistance [16].

Ecological corridors are basic elements of the landscape, and they are channels for bio-circulation and exchange between ecological sources. They are of great significance in protecting species diversity, preventing soil erosion, and improving the stability of the ecosystem [8]. The extraction methods of ecological corridors mainly include the minimum cumulative resistance (MCR) model and the circuit theory model. Compared with the MCR model, the circuit theory model focuses on the random walk characteristics of species and is able to accurately delineate the ecological protection scope by defining the specific scope and key nodes.

Combined with the ecological security patterns and the land-use status, identifying the key places that need to be protected or repaired in land-use development is of great significance. The protection of key places can better improve the pertinence of the government’s ecological protection work and is more conducive to coordinating economic development and ecological protection in highly urbanised regions. In these key places, stepping stones can be regarded as nodes in the landscape network. Species in important habitats can migrate through stepping stones. For example, in a well-connected landscape network, birds can easily find habitats and refuges that can help them forage, breed, or cross open areas [17]. At present, the threat of anti-ecological behaviour continues to increase in the Pearl River Delta (PRD) as the demands of rapid urbanisation are constantly being met. The ecological land has been destroyed, the ecological pressure is increasing and the ecological environment continues to deteriorate [18,19]. Some researchers built ecological security patterns in PRD [20,21], but their research cannot provide practical solutions for land use protection and restoration, which is not conducive to the maintenance of ecosystem stability and the improvement of landscape connectivity. These researches lack the specific identification of the specific scope of the ecological corridor and key places and also lack the analysis combined with the actual land use and development status to give the practical scope of restoration and protection.

Therefore, taking the PRD as the study area, this research aims to improve the landscape connectivity and relieve the contradiction between development and ecological protection by constructing ecological security patterns, identifying some key places, and building some ecological stepping stones. At the same time, we propose an ecological source identification process based on the resistance distance principle (RDP) to ensure the structural integrity of the ecological source. This paper aims to achieve the following goals: (1) assess the ecosystem service value through soil conservation capacity, biodiversity conservation, and the capacity of carbon fixation and identify the ecological source combined with landscape connectivity; (2) construct ecological security patterns based on circuit theory, including ecological corridors, pinch points, and barriers; (3) evaluate the conflict between ecological security patterns and current land use, then propose land-use optimisation strategies; (4) propose an ecological source extraction method based on the RDP and evaluate the feasibility of this method.

## 2. Materials and Methods

### 2.1. Study Area and Data Sources

**Figure 1:**
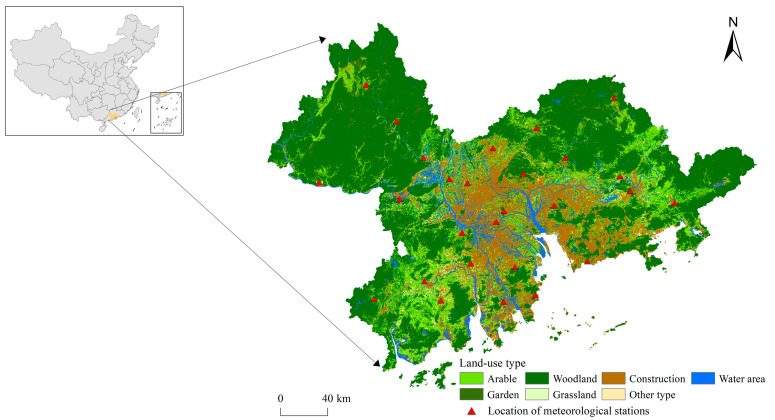
Location and land uses of the study area in 2020.

The PRD is located in southern China (21°57′–24°39′ N, 111°36′–115°42′ E), with an area of almost 53,000 km^2^, accounting for 0.55% of the total land area of China (Figure 1). The eastern, western, and northern parts of the PRD are mountainous and hilly, with rich biological resources and a low development level, while the south-central part is an alluvial plain formed by the Pearl River system, which is densely populated and highly developed. With the rapid development of the economy and urbanisation, a large amount of ecological land in this area has been artificially developed, and ecological problems have become increasingly prominent [22,23]. Under the present ecological civilisation construction background and faced with the dual pressure from land development and ecological protection, an urgent task is to identify and protect the areas of great significance to ecological security, to coordinate economic development and ecological security.

The data used in this paper are shown in Table 1. The land-use data of the PRD from 2020 (including arable, garden, woodland, grassland, construction, water area, and other types) were classified using the random forest algorithm by using Landsat 8 images on the Google Earth Engine platform. The kappa coefficient of the classification result is 0.96 indicating a high interpretation accuracy. Thus, the classification result can be used for subsequent research. The digital elevation model with a resolution of 30 m for this area was used to calculate the terrain factors in the revised universal soil loss equation (RUSLE) model. The soil erodibility factor with a resolution of 30 m, which was calculated by the second soil census, was directly substituted into the RUSLE model as the soil erodibility factor to calculate the soil conservation capacity. In addition, the carbon fixation capacity was characterised by annual net primary production (NPP) in MOD17A3H products, whose unit of measurement is g C/m^2^ [13]. Roads and railways were taken as threat sources in the construction of resistance surface. The annual average rainfall was obtained by means of kriging interpolation on the rainfall data of meteorological stations. The location of meteorological stations is shown in Figure 1. To ensure the consistent spatial resolution of the raster data, this paper resampled all raster data to 100 m × 100 m.

### 2.2. Identifying Ecological Security Patterns

**Figure 2:**
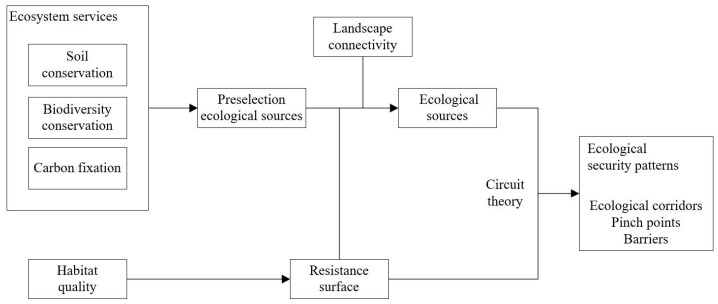
Framework for identifying ecological security patterns.

To construct ecological security patterns by adopting the paradigm that includes source identification, resistance surface construction, and corridor extraction, ecological security patterns are constructed to include ecological sources, corridors connecting ecological sources, and some key points. The specific framework of this study is shown in Figure 2. Ecological sources are not only important gathering places of ecological elements but also areas with high ecosystem service value in the region. Ecological corridors are channels with a certain width for the bio-circulation and exchange from various ecological sources. Key points include ecological pinch points and barriers. Pinch points refer to high ecological flow key nodes found in corridors, which are irreplaceable and require ecological protection as a priority, while barriers are areas that hinder the flow of bio-circulation and exchange in space and are generally regarded as conflict areas in current land development and ecological protection. After the barriers are removed, the connectivity of landscape can be improved to the greatest extent. Thus, barriers are priority areas for ecological restoration [16].

#### 2.2.1. Identifying Ecological Sources

Ecological sources are patches with high ecosystem service value, good connectivity, and great significance to regional ecological security. The rapid development of the local economy and gradual increase in construction activities, along with heavy rainfall, have resulted in a serious soil erosion problem in PRD. Habitats for wild plants and animals are especially important in areas with such high urban development such as PRD. Thus, providing living space for wildlife is also an important function of ecological source area. To achieve the goals of peak emission and carbon neutrality set by the Chinese government, carbon fixation capacity should also be listed as an important ecosystem service value. With the above conditions, data availability and other similar studies [16] taken into account, the ecosystem service values were evaluated from the following three aspects: soil conservation capacity, biodiversity conservation, and carbon fixation. In view of the different value ranges of the above three aspects, they must be normalised first, after which equal weight summation may be performed to obtain the ecosystem service value.

The soil conservation capacity was quantified by RUSLE as follows [24,25]:

(1) A=R∗K∗LS∗(1−C∗P)

where *A* is the soil conservation capacity; *R* is the rainfall erosivity factor; *K* is the soil erodibility factor; *LS* is the terrain factor; *C* is the vegetation cover management factor, which can reflect the impact of different vegetation cover conditions on soil erosion; *P* is the erosion control practise factor, which refers to the ratio of soil loss under specific conservation measures to soil loss during slope cultivation of corresponding plots before conservation measures are implemented. The *K* factor directly applied the soil erodibility factor dataset of Guangdong Province provided by the National Earth System Science Data Center, while *C* and *P* were set with reference to existing related studies [26] (Table 2).

The *R* factor was estimated by the following *R* factor estimation method based on mean annual precipitation proposed by [27] the following:

(2) $$R=587.8-1.219*Pr+0.004105*Pr^{2}$$

where *Pr* is the mean annual precipitation (mm).

The *LS* factor was estimated by the method described by [28] the following:

(3) $$LS={\left(\frac{L}{22}\right)}^{0.3}*\;{\left(\frac{\theta }{5.16}\right)}^{1.3}$$

where *L* is the slope length factor; *θ* is the slope factor. Many methods can be used to calculate the *L* factor, which in this study is directly obtained by using the built-in method of SagaGIS software [29].

On the basis of the biodiversity service evaluation method of [30] the ecological diversity service values of arable, garden, woodland, grassland, construction, water area, and other types were calculated as 0.71, 2.18, 3.26, 1.09, 0.00, 2.49, and 0.34, respectively.

The carbon fixation capacity was obtained from the annual NPP dataset (MOD17A3H), provided by the United States Geological Survey.

The areas with ecosystem service value greater than 0.709 were selected as the preselection ecological sources. The ecological sources were screened according to the resistance distance principle, and the specific process is as follows. Firstly, the maximum resistance values in all the preselection source areas were calculated. If the minimum ecological resistance between any two preselection sources was less than the maximum resistance values calculated above, then two patches will be spatially merged to form a new patch. Secondly, patches with an area smaller than 10 km^2^ were excluded. Thirdly, the importance of each patch for maintaining the probability of connectivity (*dPC*) was calculated by using Conefor software. A great *dPC* value corresponds to high connectivity importance of patches in the landscape [31]. Finally, in order from large to small, the top 50% of patches were selected as the ecological sources.

#### 2.2.2. Construction of Resistance Surface

The resistance surface reflects the resistance of ecological flow passing through different landscapes and represents the impact of landscape heterogeneity on ecological process [32]. A high habitat quality indicates a high level of biodiversity, lower interference from human activities and lower resistance to ecological processes. Therefore, the reciprocal of habitat quality was used in this paper to measure the ecological resistance. The InVEST model can be used to evaluate natural ecological services on a spatial scale, and the habitat quality module in this model can be used to evaluate the habitat quality of each grid. With reference to the existing research [33,34,35] and with the actual situation in the PRD taken into consideration, the parameters required by the model were set, including the threat factors listed in Table 3, the habitat suitability in Table 4 and the relative sensitivity of each habitat type to each threat in Table 5. Buffer zones were established for the following linear threat sources—1.5 km for railway, 1.5 km for motorway, 1 km for primary highway and 0.5 km for secondary highway—and then converted into grid threat sources [36].

#### 2.2.3. Extraction of Ecological Corridors, Pinch Points, and Barriers

In this paper, circuit theory [37,38] is used to identify ecological corridors, pinch points, and barriers in the study area. In circuit theory, electrons are regarded as individuals or genes of a species, and the landscape surface is regarded as a resistance surface. The locations with lower resistance to ecological flow are given lower resistance values; otherwise, they are given higher resistance values. Following this, the resistance distance is calculated, and the threshold is set to extract the direction and spatial range of the ecological corridor in the landscape. Next, the current is used to simulate the migration and diffusion processes of species or biological flow on the landscape surface. In this paper, the all-to-one model is used to perform the simulation. During this process, a source is connected to a power supply, and other ecological sources are grounded. Consequently, the current density between sources could be calculated, which can represent the probability of migration and diffusion of biological flow [39]. Pinch points are the regions with high current density, which is the key area in the migration and diffusion of biological flow. Barriers are areas that can greatly improve the connectivity of ecological sources after some resistance in the landscape has been removed and can be regarded as key areas for landscape restoration.

We set the threshold value of extraction ecological corridors based on the assumption that the investment in protection of ecological corridors can cover only 20% of the whole study area [28]. In the extraction process of pinch points and barriers, the cumulative current and the expected reduction value of least-cost distance per unit distance were calculated firstly through Pinchpoint Mapper and Barrier Mapper in Linkage Mapper toolkit. Then, we divided them into five classes by using Jenks. Finally, the maximum class of cumulative current and the maximum class of the expected reduction value of least-cost distance per unit distance were regarded as the pinch points and barriers, respectively.

## 3. Results

### 3.1. Ecological Sources and Resistance Surface

**Figure 3:**
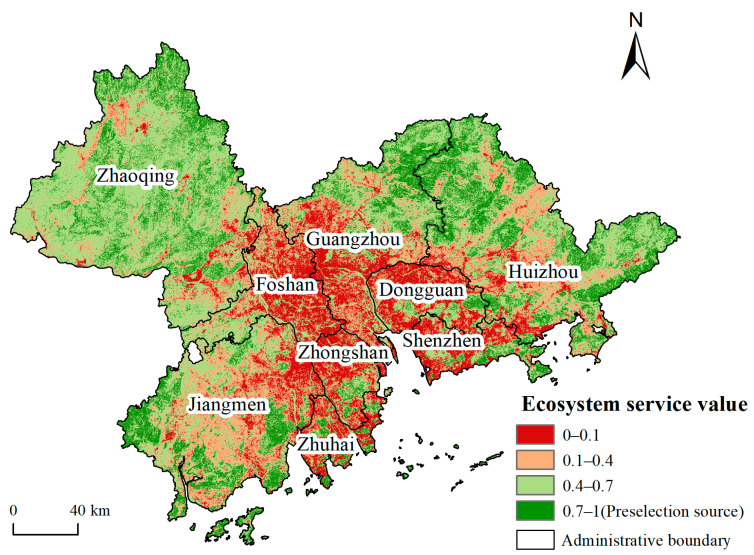
Spatial distribution of ecosystem service values and preselection ecological sources.

**Figure 4:**
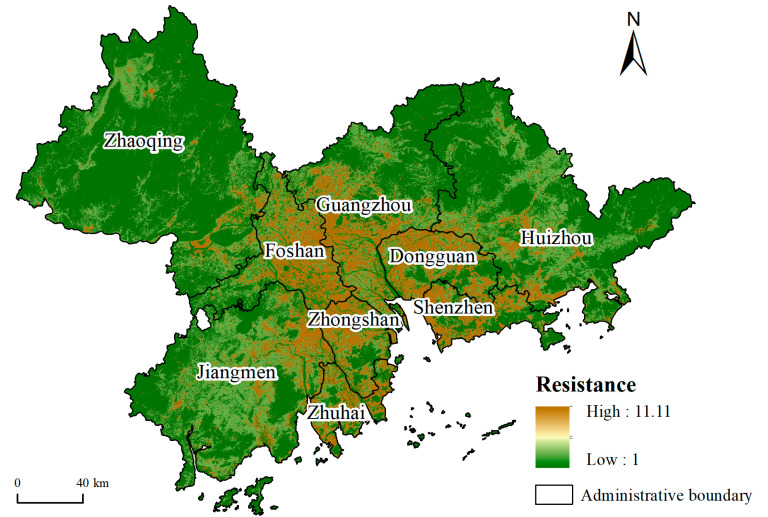
Resistance surface.

**Figure 5:**
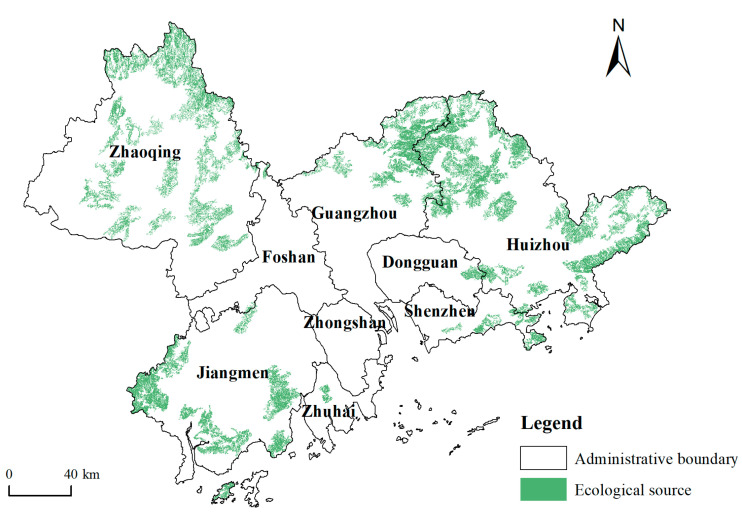
Spatial distribution of ecological sources.

The ecosystem service value of each grid unit in the study area was evaluated by the method described in Section 2.2.1, and the areas with values greater than 0.709 were taken as the preselection sources (Figure 3). The method described in Section 2.2.2 was then used to obtain the resistance surface (Figure 4). Through the statistical overlay of the preselection sources and resistance surface, the maximum ecological resistance in the preselection sources was obtained, which was 1.017. Therefore, in this paper, if the minimum ecological resistance between any two preselection sources was less than 1.017, then they were merged spatially into a new ecological source. After being screened by area and connectivity, 46 ecological sources with a total area of 6859.47 km^2^ were finally obtained, accounting for 12.94% of the total area of the study area (Figure 5).

Overall, obvious differences exist in the ecological source areas of different cities in the PRD. Huizhou has the largest ecological source area, accounting for 36.33% of the total area of regional ecological sources. A large number of ecological sources are also present in Zhaoqing, Jiangmen, and Guangzhou, accounting for 28.00%, 18.32%, and 12.84%, respectively. The ecological source areas of the other five cities are very small, accounting for only 4.51% of the total ecological source areas, which reflects the high degree of development in the plain of PRD. Significantly, most of the plain areas have been developed in PRD, and the ecological security has been greatly affected, especially in Foshan, Zhongshan, Dongguan, Shenzhen, and Zhuhai, located on either side of the Pearl River estuary. Most of the land in these cities is flat and has been developed and utilised. However, in these cities, most ecological lands are small and not contiguous, which makes forming a large-scale ecological source with strong radiation difficult. The large ecological sources of more than 200 km^2^ in the study area are mainly distributed in Shimen National Forest Park; Nankun, Luofu, and Xiangtou Mountains in northeastern Guangzhou and northwestern Huizhou; Lianhua Mountain in eastern Huizhou; Gudou, Dalongdong, Junzi, and Tianlu Mountains in Jiangmen; and Luoke and Dinghu Mountains in Zhaoqing.

The resistance value can directly show the degree of human disturbance to the ecological environment. A high value corresponds to low habitat quality. In other words, human disturbance is more serious, and its influence on the biological process is greater. The ecological resistance value of each grid unit in the study area ranged from 1 to 11.11, with an average resistance of 3.82. The average resistance value of Dongguan was the highest (6.39), followed by that of Zhongshan (6.22), while the value of Zhaoqing was the lowest (1.56). Figure 4 shows that the resistance value in the central plain of the study area was significantly higher than that in the surrounding mountainous areas.

### 3.2. Ecological Security Patterns

**Figure 6:**
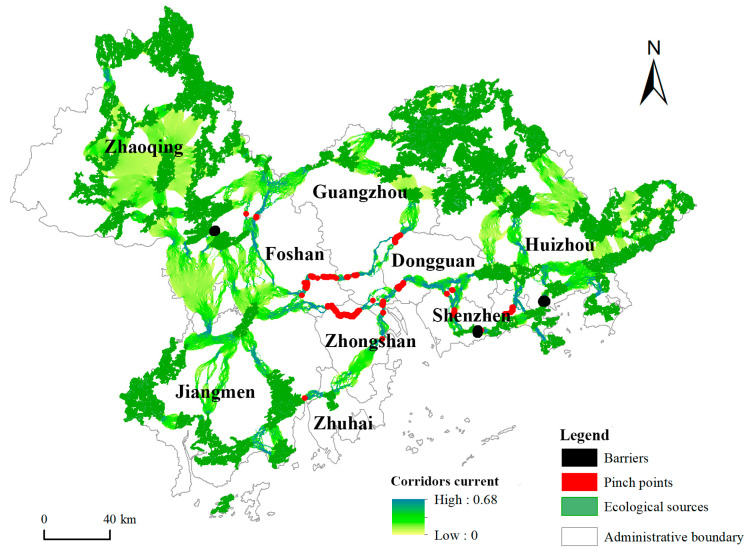
Ecological security patterns in the PRD region.

In this paper, 84 ecological corridors, 90 ecological pinch points, and three barriers were identified (Figure 6). The total length of ecological corridors is 2028.80 km, and the areas of ecological pinch points and barriers are 10.90 and 29.57 km^2^, respectively. The ecological corridors in the study area are generally spider-like in shape. They closely connect the ecological sources in the area and are of great significance to the ecological flow in the area. However, significant spatial differences are present in the ecological corridors, with the ecological corridors in most areas of Zhaoqing and Huizhou, southwestern Jiangmen, and northern Guangzhou having a short distance, a large width, and a low current density. Among the large ecological sources in these areas, a certain number of small and medium-sized ecological sources are present, which act as stepping stones and connect the large sources into a network. This finding indicates that these areas are less disturbed by human beings and have lower resistance to ecological flow. In contrast, the ecological corridors in most areas of Foshan, Dongguan, Shenzhen, and Zhongshan, southeastern Zhaoqing, and southern Guangzhou are relatively long and narrow, indicating that these areas lack such stepping stones. Thus, the ecological flow between the source areas must travel a long distance. What exacerbates the situation is that the ecological corridors do not have enough width; thus, these areas are easily interfered with by humans. In addition, the current density in these areas is generally high, and a large number of ecological pinch points are distributed, which is the key protection area.

The pinch points are the regions with high current density in the migration and diffusion process of biological flow. They are mainly distributed in the ecological corridors throughout the Pearl River estuary, which is mainly covered by the Guangdong government’s Wanli Bidao river protection project. They are generally distributed in a zonal concentration. Zhongshan has the largest distribution area, with a total area of 4.05 km^2^, accounting for 37.15% of the total area of pinch points. A certain number of ecological pinch points are found in southeastern Foshan, southern Guangzhou, Dongguan, and Shenzhen. Barriers are distributed throughout Zhaoqing, Shenzhen, and Huizhou, all of which are urban areas between two adjacent ecological sources. In addition, a large amount of construction land is blocked between ecological corridors in adjacent sources, which significantly increases the difficulty of ecological flow, thereby forming barriers to the key restoration areas.

## 4. Discussion

### 4.1. Optimisation Strategy for Ecological Security Patterns

**Figure 7:**
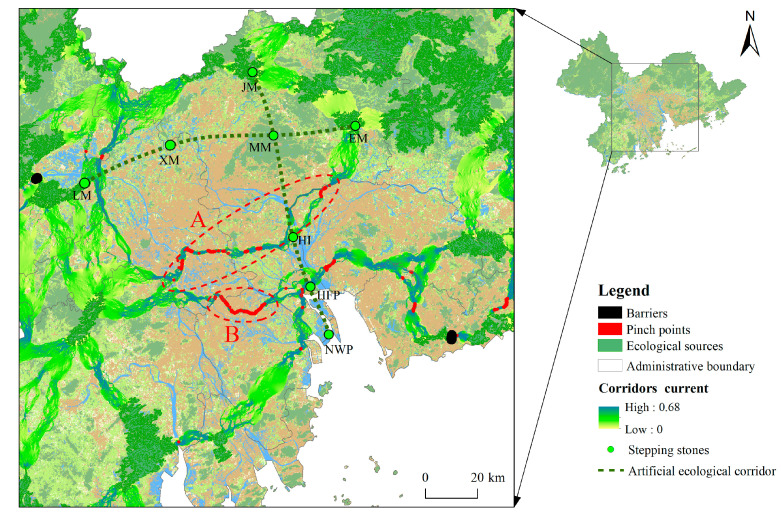
Ecological security patterns and ecological network planning map in key areas.

The identified ecological pinch points are mainly concentrated in the built-up area in the central part of the study area (Figure 7). Ecological pinch points are mainly distributed in a banded pattern in two ecological corridors, which are defined as A and B for convenient description. Overlay analysis with land cover shows that the ecological corridors in areas A and B overlapped with the existing rivers. Area A passes through the Xijiang River, Ganzhuxi River, Shunde Waterway, Humen Waterway, and Dongjiang River, while Area B passes through the Jiya Waterway, Huangshali Waterway, and Shanghengli Waterway. Both areas A and B are key sections of the ecological corridor connecting ecological sources on either side of the Pearl River estuary. In areas with high urbanisation and high intensity of human activities, rivers are of great significance for ecological flow. Therefore, the protection and restoration of the above rivers will aid in improving the ecological connectivity of river corridors, such as improving river water quality through river dredging and water treatment and improving ecological functions by building green corridors on both sides of rivers. With the exception of pinch points in areas A and B, other pinch points coincide with construction land or unused land. For developed construction land, green construction should be emphasised on the basis of fully considering the current situation of land use. For unused land, priority should be given to the construction of ecological parks to ensure the ecological function of pinch points as much as possible [28].

Three barriers identified in this paper are located along the narrow passages of Dinghu Mountain–Lanke Mountain, Yinhu Mountain–Wutong Mountain, and Bijia Mountain–Yaji Mountain. All of these are all built areas between adjacent ecological sources. The areas where the barriers are located all consist of urban construction land. Thus, ecological restoration through removal is not realistic. Local vegetation should be introduced as much as possible, and the vegetation coverage of the barriers should be improved within the scope of practical conditions to enhance the ecological function. This finding also suggests that in future land development planning, designating some continuous areas as nature reserves will not be sufficient, and narrow corridors between ecological sources should also be listed as key protected areas.

The ecological security pattern identification results analysed in this study are basically similar to the results of studies in similar research areas [20]. The ecological security patterns identified in this study suggest the poor network connectivity of ecological sources on both sides of the Pearl River estuary. Therefore, creating some ecological stepping stones is necessary to enhance regional corridor connectivity. In the research areas, Gull Island (GI) and Maofeng Mountain (MM) can be used as important ecological stepping stones for ecological protection, which is basically consistent with the current planning of GI and MM, with ecological leisure tourism as the development goal. At the estuary of the Pearl River, Nansha Wetland Park (NWP) and Huangshanlu Forest Park (HFP) are located at the intersection of the existing horizontal natural ecological corridors and the main stream of the Pearl River. These areas provide habitats for a large number of wild animals and play a major role in maintaining biodiversity, purifying the air and regulating the climate [40], thus making them suitable as important ecological stepping stones. While the northern part of Foshan City and the Baiyun District of Guangzhou City still retains a certain amount of ecological land, ecological land in this area has been continuously destroyed by humans in recent years [41]. Therefore, a necessary step is to implement ecological protection in this area in the future and promote the improvement of the ecological functions of the remaining ecological land. Among them, Xianggangling Mountain (XM), which has a large area and high ecosystem service value, can be used as an ecological stepping stone that connects the ecological sources of the east and west banks of the Pearl River. Combined with the identified ecological security patterns of the study area, the existing nature reserves and actual land use, this paper proposes the establishment of the east-west artificial ecological corridor of Lanke Mountain (LM)–Xianggangling Mountain (XM)–Maofeng Mountain (MM)–Erlong Mountain (EM) and the north-south artificial ecological corridor of Jizhen Mountain (JM)–Maofeng Mountain (MM)–Gull Island (GI)–Huangshanlu Forest Park (HFP)–Nansha Wetland Park (NWP). This approach will form a fence-shaped corridor construction blueprint with the existing corridors to enhance the network connectivity of ecological corridors on both sides of the Pearl River estuary, thereby providing a scientific reference for ensuring the ecological safety of the region.

### 4.2. Feasibility Analysis of Ecological Source Identification Method Based on the Resistance Distance Principle

**Figure 8:**
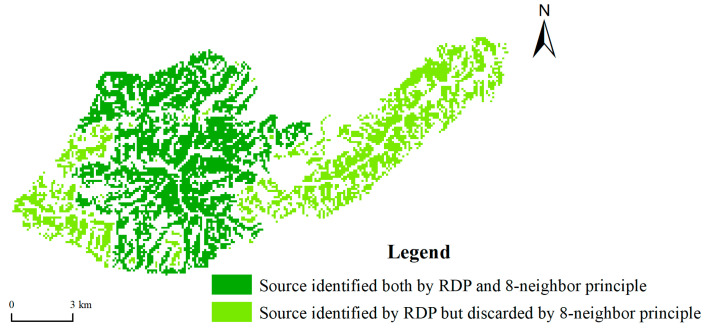
Example of identification results based on RDP and eight-neighbourhood principles.

The identification of ecological sources is the first key step to constructing ecological security patterns, and the integrity of identification results determines the rationality of ecological security patterns that are finally obtained. At present, using the comprehensive evaluation method to identify ecological sources often requires an area screening process; namely, small ecological source areas will be excluded, with only large patches remaining. However, this process involves the issue of how to define the scope of the source area. At present, most scholars use the eight-neighbourhood principle to define the source scope by default [13,16,42]. However, according to this principle, the source patches obtained by the comprehensive evaluation method are relatively broken, and large quantities of sources are discarded in many cases. As shown in Figure 8, the abandoned large areas on the left and right in fact have a short spatial distance from the preserved source, and the ecological resistance between them is low. Thus, they should be regarded as one source. If the left and right parts are abandoned, then the integrity of the source is destroyed. The method based on the proposed RDP can resolve the above issues. RDP regards the maximum internal resistance of all preselection sources as the resistance threshold and spatially merges the source groups whose ecological resistance between the sources is less than the threshold, thus obtaining a new ecological source. According to statistics, the ecological source extracted by this method is 1978.58 km^2^ greater than that extracted by the traditional eight-neighbourhood principle. This approach reduces the loss of source area by 28.84% and greatly improves the integrity of source identification results.

## 5. Conclusions

In this paper, an ecological source identification method based on the RDP was proposed to improve the integrity of the ecological source identification results. On the basis of circuit theory, ecological security patterns were constructed, including ecological corridors, ecological pinch points, and barriers. Combined with the actual land use, future land use optimisation strategies based on ecological security patterns in the PRD region were proposed. The main conclusions are as follows:

(1) Ecological security patterns of the PRD include 46 ecological sources, 84 ecological corridors, 90 pinch points, and 3 barriers. The difference in the spatial distribution of the ecological sources is remarkable. A large number of large ecological sources are distributed in the mountainous areas surrounding the study area, while the ecological sources on both sides of the Pearl River estuary are few and have a small area. The ecological corridors are generally spider-like in shape, but they are long and narrow in the central plains of the PRD with many ecological pinch points, thus indicating that the ecological security in this area is facing great pressure. Therefore, those areas are key protected areas. The barriers are mainly distributed between ecological corridors adjacent to the source areas, which are the key restoration areas.
(2) In highly urbanised areas, ecological pinch points are concentrated on existing rivers, thus indicating that riparian corridors in highly urbanised areas are of utmost importance for the ecological process. The overall ecological function of the river can be improved by improving river water quality through river dredging and water treatment or by constructing green corridors on both sides of rivers. For pinch points that overlap with unused land and construction land, priority should be given to the construction of ecological parks, and greening construction should be emphasised to enhance the ecological function of the pinch points.
(3) Combined with the construction ecological security patterns of the study area and the existing nature reserves, this paper proposes the establishment of the east-west artificial corridor of ’Lanke Mountain—Xianggangling Mountain—Maofeng Mountain—Erlong Mountain’ and the north-south artificial corridor of ’Jizhen Mountain—Maofeng Mountain—Gull Island—Huangshanlu Forest Park—Nansha Wetland Park’ to form a fence-shaped corridor construction blueprint with the existing corridors, which can enhance the network connectivity of ecological corridors on both sides of the Pearl River estuary, thereby ensuring the overall ecological security of the region.
(4) Compared with the ecological source identification method based on the eight-neighbourhood principle, the method based on the proposed RDP can reduce the impact of patch space fragmentation in preselection source areas, greatly improving the integrity of the source extraction results and ensuring reasonable construction results of ecological security patterns.

Limited by the availability of data and difficulty in quantification, some factors that may affect ecological security patterns, such as the pathological degree of ecosystem risk, the pollution degree of water resources, and the blocking effect of roads, are not involved in this model. In addition, the setting of some parameters in the construction of ecological security patterns, such as the minimum area threshold of the source area, the extraction threshold of corridor resistance, and the extraction threshold of pinch points and barriers, requires further improvement. Further research on these aspects will be conducted in the future to obtain more realistic research conclusions.

## Figures and Tables

**Table 1:** Information of basic data.

Data Category	Data name	Time	Resolution	Data Source
Remote sensing data	Landsat8	2020	30 m	Google Earth Engine
Basic geographic data	Administrative boundary	2015	-	National Catalogue Service for Geographic Information (http://www.webmap.cn/) (accessed on 1 August 2021).
	Road	2020	-	OpenStreetMap (https://www.openstreetmap.org/) (accessed on 1 August 2021).
	Railway	2020	-	
Natural environment data	DEM	2009	30 m	Geospatial Data Cloud (http://www.gscloud.cn/) (accessed on 1 August 2021).
	Soil erodibility factor (K factor in RUSLE)	2020	30 m	National Earth System Science Data Center, National Science and Technology Infrastructure of China (http://www.geodata.cn) (accessed on 1 August 2021).
	NPP	2020	500 m	United States Geological Survey (https://lpdaac.usgs.gov/products/mod17a3hgfv006/) (accessed on 1 August 2021).
Meteorological data	Precipitation data of meteorological stations	1981–2010	-	China Meteorological data service center (http://data.cma.cn/) (accessed on 1 August 2021).

**Table 2:** C and P factor of different land-use types.

Land-Use Types	C	P
Arable	0.12	0.15
Garden	0.15	0.15
Woodland	0.02	1
Grassland	0.15	1
Construction	0	0
Other Type	0	1
Water Area	0	0

**Table 3:** Threat factor parameter settings.

Threat	Max Distance	Weight	Decay Type
Arable	6	0.7	Exponential
Garden	8	0.8	Exponential
Construction	11	0.95	Exponential
Other Type	3	0.4	Linear
Railway	9	0.9	Exponential
Motorway	10	1	Exponential
Primary-highway	8	1	Linear
Secondary-highway	5	0.75	Linear

**Table 4:** Habitat suitability of each land use/land cover type.

Land-Use Types	Arable	Garden	Woodland	Grassland	Construction	Other Type	Water Area
Habitat Score	0.5	0.6	1	0.55	0	0.1	0.9

**Table 5:** Sensitivity score of habitat types to threat factors.

Land-Use Types	Threat Factors							
	Arable	Garden	Construction	Other Type	Railway	Motorway	Primary-Highway	Secondary-Highway
Arable	0.3	0.35	0.5	0	0.1	0.25	0.28	0.22
Garden	0.35	0.4	0.6	0	0.1	0.15	0.18	0.2
Woodland	0.4	0.5	0.6	0.05	0.1	0.1	0.12	0.18
Grassland	0.35	0.3	0.6	0	0.25	0.25	0.28	0.29
Construction	0.5	0.5	0.8	0	0.3	0.3	0.32	0.31
Other Type	0.1	0.2	0.7	0.5	0.25	0.2	0.2	0.2
Water Area	0.5	0.5	0.8	0	0.4	0.4	0.35	0.3

## Data Availability

The data presented in this study are available from author upon reasonable request.
